# High KLF4 level in normal tissue predicts poor survival in colorectal cancer patients

**DOI:** 10.1186/1477-7819-12-232

**Published:** 2014-07-24

**Authors:** Ha-young Lee, Joong Bae Ahn, Sun Young Rha, Hyun Cheol Chung, Kyu Hyun Park, Tae Soo Kim, Nam Kyu Kim, Sang Joon Shin

**Affiliations:** 1 Division of Hematology and Oncology, Department of Internal Medicine, Dongnam Institute of Radiological and Medical Sciences, Jwadong-gil 40, Jangan-eup, Gijang-gun, Busan 619-953, South Korea; 2 Department of Internal Medicine, Yonsei Cancer Center, Yonsei University College of Medicine, 50 Yonsei-ro, Seodaemun-gu, Seoul 120-752, South Korea; 3 Cancer Metastasis Research Center, Yonsei Cancer Center, Yonsei University College of Medicine, 50 Yonsei-ro, Seodaemun-gu, Seoul 120-752, South Korea; 4 Department of Surgery, Yonsei Cancer Center, Yonsei University College of Medicine, 50 Yonsei-ro, Seodaemun-gu, Seoul 120-752, South Korea

**Keywords:** KLF4, Prognostic marker, Colorectal cancer

## Abstract

**Background:**

*Krüppel-like factor 4 (KLF4)* is involved in many important cellular processes such as growth, development, differentiation, proliferation, and apoptosis. The purpose of this study was to determine the significance of *KLF4* in both tumors and normal tissues of patients with colorectal cancer (CRC).

**Methods:**

Between January 2003 and June 2005, 125 patients with CRC receiving treatment at the Yonsei Cancer Center were selected. We examined the mRNA level of the *KLF4* gene in primary CRC specimens and matched normal colon tissues using real-time RT-PCR. Correlation of survival with clinicopathological parameters, including *KLF4* level, was investigated with univariate and multivariate analyses.

**Results:**

CRC tissue had a significantly lower *KLF4* level when compared with matched normal tissues (*KLF4* in tumors: 2007 ± 1531 copies/μl, *KLF4* in normal tissues: 6586 ± 2834 copies/μl; *P* <0.0001). However, there was a correlation between the *KLF4* level in tumors and normal tissues. Patients with a high *KLF4* level in matched normal tissues were more likely than those with a low *KLF4* level to develop recurrence and had poorer overall survival (*P* = 0.005). Therefore, the *KLF4* level in the normal tissue of individuals was associated with prognosis of individuals.

**Conclusions:**

Our data suggest that *KLF4* mRNA expression level in normal tissues and tumors may be a useful prognostic marker in patients with CRC.

## Background

Worldwide, colorectal cancer (CRC) is the fourth most commonly diagnosed cancer in men and third in women. CRC is also the third most common cause of cancer-related death [1]. Despite advances in diagnosis and treatment of CRC, it remains a disease with high morbidity and mortality. Assessment of prognosis through tumor node metastasis (TNM) staging systems remains inappropriate due to the considerable diversity and heterogeneity among tissues of the same stage. Therefore, it is a necessary to establish novel prognostic markers for cancer recurrence and survival to improve treatment for individual patients.

*Krüppel-like factors (KLFs)* are a family of evolutionarily conserved mammalian zinc-finger transcription factors named for their homology with Krüppel, a *Drosophila melanogaster* protein [2]. It is now well established that *KLFs* are involved in many important cellular processes such as growth, development, differentiation, proliferation, and apoptosis [3-5]. *KLF4* (also called gut-enriched *KLF* or *GKLF*) was one of the first *KLF* family members identified [6,7]. In addition to regulating many important physiological processes, *KLF4* has been shown to play a role in pathologic conditions such as cancer and inflammation [8-13]. More recently, *KLF*4 was shown to play a crucial role in the reprogramming of somatic cells into induced pluripotent stem cells [10]. *KLF4* is primarily expressed in postmitotic, terminally differentiated epithelial cells and is stimulated following p53-dependent DNA damage [14,15].

*KLF4* expression is activated in breast cancer and oropharyngeal squamous cell carcinoma [16,17], whereas its expression in colorectal, gastric, and lung cancer is reduced relative to normal tissues [13,18-22]. Thus, the expression and role of *KLF4* may vary according to cancer location. Several studies have shown that *KLF4* mRNA and protein expression is reduced in human colorectal cancer [13,18,20,22]. Moreover, the level of *KLF4* expression is significantly decreased in familiar and/or sporadic colonic adenomas and carcinomas when compared with normal colonic tissues [13]. Taken together, these findings suggest that downregulation of *KLF4* protein expression in the colon may contribute to cellular hyperproliferation and malignant transformation. Until recently, the principal focus in cancer research has mostly been the malignant cell itself. However, we now know that tumor growth is not determined by malignant cells alone, but that the tumor microenvironment has a major impact on cancer growth, progression, and prognosis [23]. In this study, we examined the *KLF4* mRNA level in colorectal tumors and normal tissues and evaluated the clinical significance of *KLF4* expression on prognosis in CRC patients.

## Methods

### Tissue samples

A total of 125 paraffin-fixed colorectal cancer and matched normal tissue samples were obtained between January 2003 and June 2005 from the Yonsei Cancer Center, Severance Hospital (South Korea). All tumors and matched normal tissues were obtained from surgical specimens of patients with CRC. The matched normal tissues were at least 2 cm away from the edge of corresponding tumors. This study was approved by the Severance Hospital Institutional Review Board, which waived the requirement for informed consent.

### Real-time reverse transcription-polymerase chain reaction analysis

Total RNA was isolated using the TRIzol™ method (Invitrogen, Carlsbad, California, United States), and first-strand cDNA was synthesized with Moloney murine leukemia virus reverse transcriptase (Promega, Madison, Wisconsin, United States). Single-strand cDNA was used as a template for subsequent PCR. Two microliters of cDNA from each tissue were used for the real-time reverse transcription-polymerase chain reaction (RT-PCR) assay. Templates were amplified using QuantiTect SYBR Green PCR kit (QIAGEN, Valencia, California, United States). Primer (Proligo, Singapore, Singapore) sequences used in RT-PCR were: 5'-GGCAAAACCTACACAAAGAG-3' and 5'-GTAGTGCCTGGTCAGTTCAT-3'. PCR was initiated at 95°C for 15 minutes to activate the HotstarTaq DNA polymerase (QIAGEN, Hilden, Germany), and then amplified for 35 cycles at 95°C for 20 s, 50°C for 30 s, and 72°C for 45 s on a Rotor Gene 2072D real-time PCR machine (Corbett Research, New South Wales, Sydney, Australia). The amplified fluorescence signal in each specimen was measured at the late extension step of each cycle. To quantify each gene, 10-fold dilution of human genomic DNA was used (Promega).

### Data analysis

**Figure 1:**
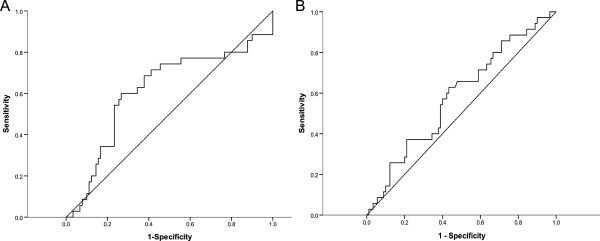
**ROC curve. (A)** ROC curve of *tKLF4* (AUC 0.61). **(B)** ROC curve of *nKLF4* (AUC 0.58).

All statistical analyses were performed using SPSS for Windows software, version 18.0 (SPSS Inc, Chicago, Illinois, United States). We used a paired t-test to compare the *KLF4* level between tumors and matched normal tissues. The *tKLF4* (*KLF4* level in tumors) cutoff level was set to 2150 pg/dl (Area under the curve (AUC) 0.61, sensitivity 60%, specificity 73%), and the *nKLF4* (KLF4 level in normal tissues) cutoff level was set to 7969 pg/dl (AUC 0.58, sensitivity 40%, specificity 65%) in a Receiver operating characteritsitc (ROC) curve (Figure 1). The patients were classified into two groups using the *KLF4* cutoff levels. The *χ*^2^ test was applied to find the correlation between the *KLF4* level and clinicopathologic parameters. A binary logistic model was used in the multivariate analysis. Overall survival (OS) was measured from the date of the CRC diagnosis to the date of death. Disease-free survival (DFS) was defined as the time between the diagnosis of CRC and recurrence of the disease. Survival plots were estimated using the Kaplan-Meier method, and differences in survival distributions were evaluated using the log-rank test. In multivariate analysis, Cox proportional hazards models were used to analyze the effect of specified risk factors on OS. *P* < 0.05 was considered to be statistically significant.

## Results

### Patient characteristics

Patient characteristics are shown in Table 1. The median age of patients was 62 years (range 35 to 83 years), and 65.6% were male. On first diagnosis, 16.0% of the patients were stage I (n = 20), 24.8% were stage II (n = 31), 44.0% were stage III (n = 55), and 15.2% were stage IV (n = 19). The anatomic site of the tumors was defined as right colon (ascending colon cancer, transverse colon cancer), left colon (descending colon cancer, sigmoid colon cancer, rectosigmoid colon cancer), and rectum (rectal cancer). Tumors were distributed with 19.2% (n = 24) on the right colon, 36.0% (n = 45) on the left colon, and 44.8% (n = 56) on the rectum. A median of 20 lymph nodes (range 3 to 64) were taken and in only 12.8% (n = 16) of patients, less than 12 lymph nodes were taken. In 56.0% (n = 70) of patients, metastasis to the lymph nodes was shown. Curative resection was performed in 90.4% (n = 113) of patients, and palliative surgery was performed in 9.6% (n = 12) of patients. Among patients with stage IV (n = 19), 36.8% (n = 7) received curative resection (primary tumor resection and metastasectomy) and 63.2% (n = 12) received palliative surgery. The median follow-up period was 68.7 months (range 0.4 to 96.3 months), and the median survival of surviving patients was 94.8 months (range 72.8 to 102.6 months). Thirty-five patients died with a median survival of 29.8 months (range 3.0 to 75.1 months), and the overall five-year survival was 78.4% (98 out of 125).

**Table 1:** Patient characteristics

	**Number of patients (%)**
Total patients	125
Age in years (median)	35-83 (62.0)
≤60	50 (40.0)
≥60	75 (60.0)
Sex	
Male	82 (65.6)
Female	43 (34.4)
Positive nodes	
absent	55 (44.0)
present	70 (56.0)
Stage	
I	20 (16.0)
II	31 (24.8)
III	55 (44.0)
IV	19 (15.2)
Primary lesion	
Right colon	24 (19.2)
Left colon	45 (36.0)
Rectum	56(44.8)
Histology	
Well differentiated	16 (12.8)
Moderately differentiated	100 (80.0)
Poorly differentiated	5 (4.0)
Mucinous	4 (3.2)
Blood/lymphatic vessel invasion	
Absent	109 (87.2)
Present	16 (12.8)
Surgery	
Curative	113 (90.4)
Palliative	12 (9.6)

### Factors associated with *KLF4* expression in normal and tumor tissues

**Figure 2:**
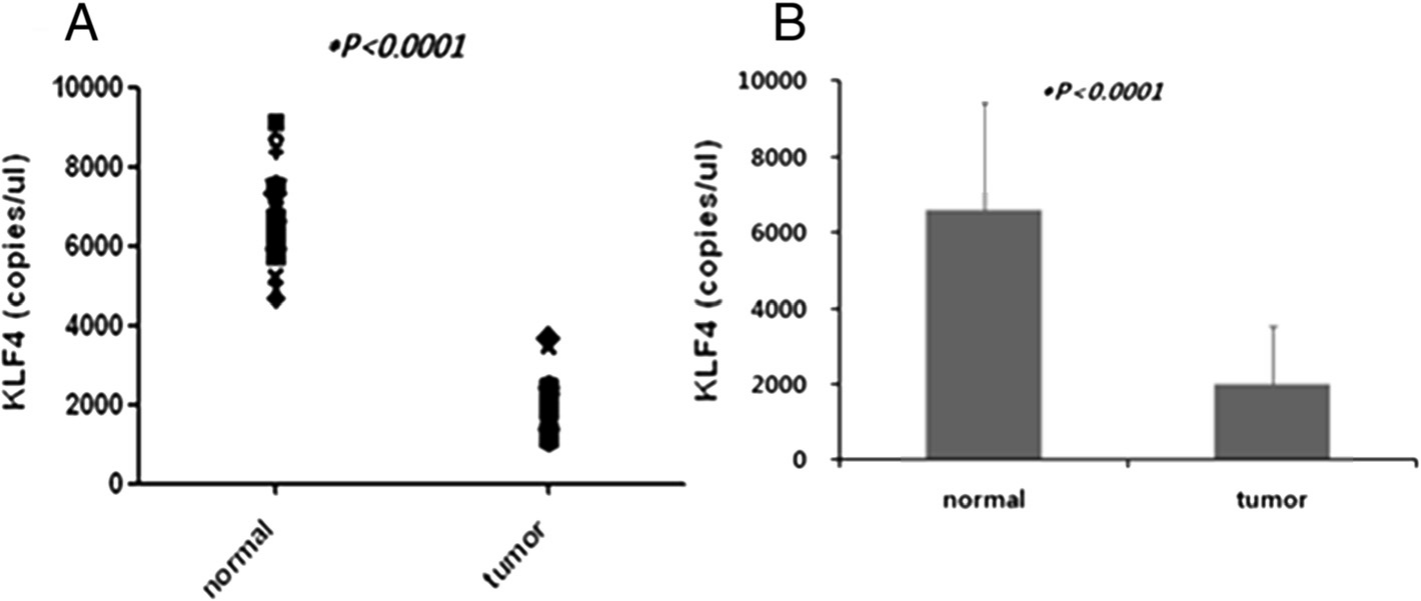
***KLF4 *expression in CRC and matched normal tissues. (A)***KLF4* mRNA expression levels were significantly decreased in CRC tissues compared with matched normal tissues. **(B)** Bar graph of KLF4 mRNA expression levels.

**Figure 3:**
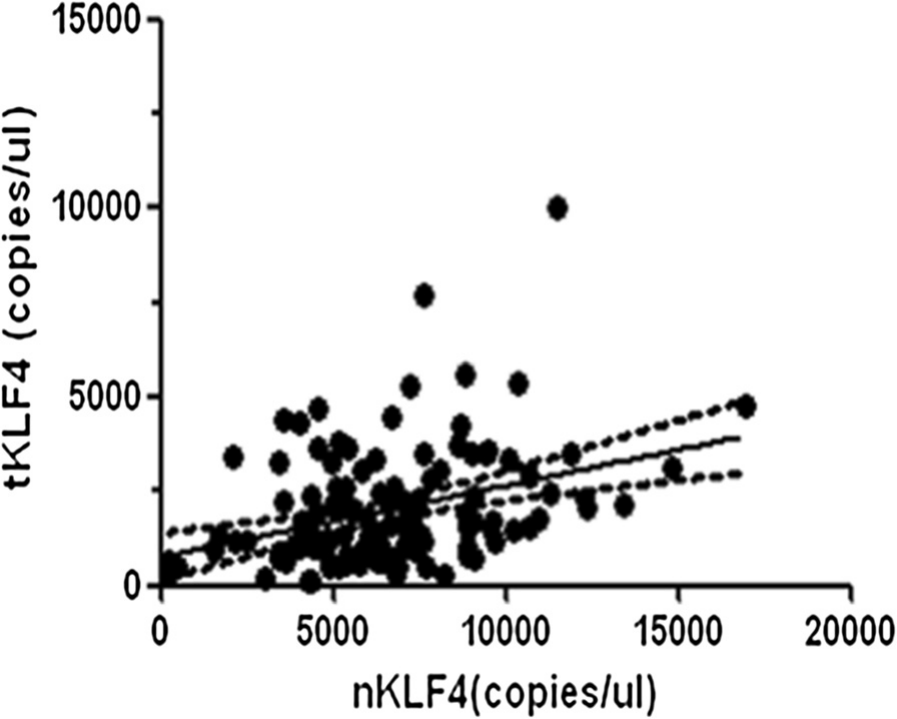
**Correlation analysis of *nKLF4 *and *tKLF4 *expression.** (r^2^ = 0.349, *P* <0.0001).

*KLF4* mRNA expression levels were significantly decreased in CRC tissues compared with matched normal tissues (*tKLF4*: 2007 ± 1531 copies/μl, *nKLF4*: 6586 ± 2834 copies/μl) (*P* <0.0001) (Figure 2). However, there was a correlation between the *nKLF4* and *tKLF4* level (*P* <0.0001) (Figure 3). In multivariate and univariate analysis, *tKLF4* was significantly associated with the tumor stage (univariate analysis: *P* = 0.031, multivariate analysis: *P* = 0.044) (Table 2). *nKLF4* level had a significant relationship with age and anatomic distribution in a multivariate logistic model that took into account age, stage, gender, lymph nodes metastasis, and anatomic distribution (*P* = 0.024, *P* = 0.046: right and left colon, *P* = 0.027: right colon and rectum, respectively) (Table 3). No significant differences were observed among gender, differentiation, or lymph node status.

**Table 2:** ***KLF4 *** **expression in CRC and paired normal tissue**

**Variable**	**tKLF4**		***P *** value^*^	**nKLF4**		***P *** value^*^
	**<2150**	**≥2150**		**<7969**	**≥7969**	
**Age** (years)						
<60	36 (72.0%)	14 (28.0%)		42 (84.0%)	8 (16.0%)	
≥60	44 (58.7%)	31 (41.3%)	0.128	51 (68.0%)	24 (32.0%)	0.045
**Gender**						
Male	49 (60.5%)	32 (39.5%)		59 (72.8%)	22 (27.2%)	
Female	31 (70.5%)	13 (29.5%)	0.268	34 (77.3%)	10 (22.7%)	0.588
**Stage**						
I-III	72 (67.9%)	34 (32.1%)		79 (74.5%)	27 (25.5%)	
IV	8 (42.1%)	11 (57.9%)	0.031	14 (73.7%)	5 (26.3%)	0.938
**LN metastasis**						
+	46 (64.8%)	25 (35.2%)		55 (77.5%)	16 (22.5%)	
−	34 (63.0%)	20 (37.0%)	0.833	38 (70.4%)	16 (29.6%)	0.368
**Anatomic distribution**						
Right	14 (58.3%)	10 (41.7%)		22 (91.7%)	2 (8.3%)	
Left	33 (73.3%)	12 (26.7%)		32 (71.1%)	13 (28.9%)	
Rectum	33 (58.9%)	23 (41.1%)	0.264	39 (69.9%)	17 (30.4%)	0.096

^*^P value estimated by Chi-square *χ*2 test.

**Table 3:** **Multivariate analysis of *nKLF4 *** **expression**

**Variable**	**Estimated coefficient**	**Estimated standard error**	**Wald X2 statistics**	***P *** value^*^	**Estimated odds ratio**	**Confidence interval for odds ratio**
Stage (I-III, IV)	0.108	0.611	0.031	0.860	1.114	(0.336-3.685)
Age in years (<60, ≥60)	1.087	0.482	5.076	0.024	2.965	(1.152-7.631)
Gender (M, F)	−0.314	0.471	0.443	0.505	0.731	(0.290-1.840)
LN metastasis (+,−)	0.649	0.459	1.994	0.158	1.913	(0.778-4.705)
Anatomic distribution (right, left colon)	1.644	0.826	3.964	0.046	5.178	(1.026-26.135)
Anatomic distribution (right colon, rectum)	1.801	0.815	4.880	0.027	6.054	(1.225-29.916)

^*^P value estimated by binary logistic model including all available covariates.

### Implication of KLF4 on survival

**Figure 4:**
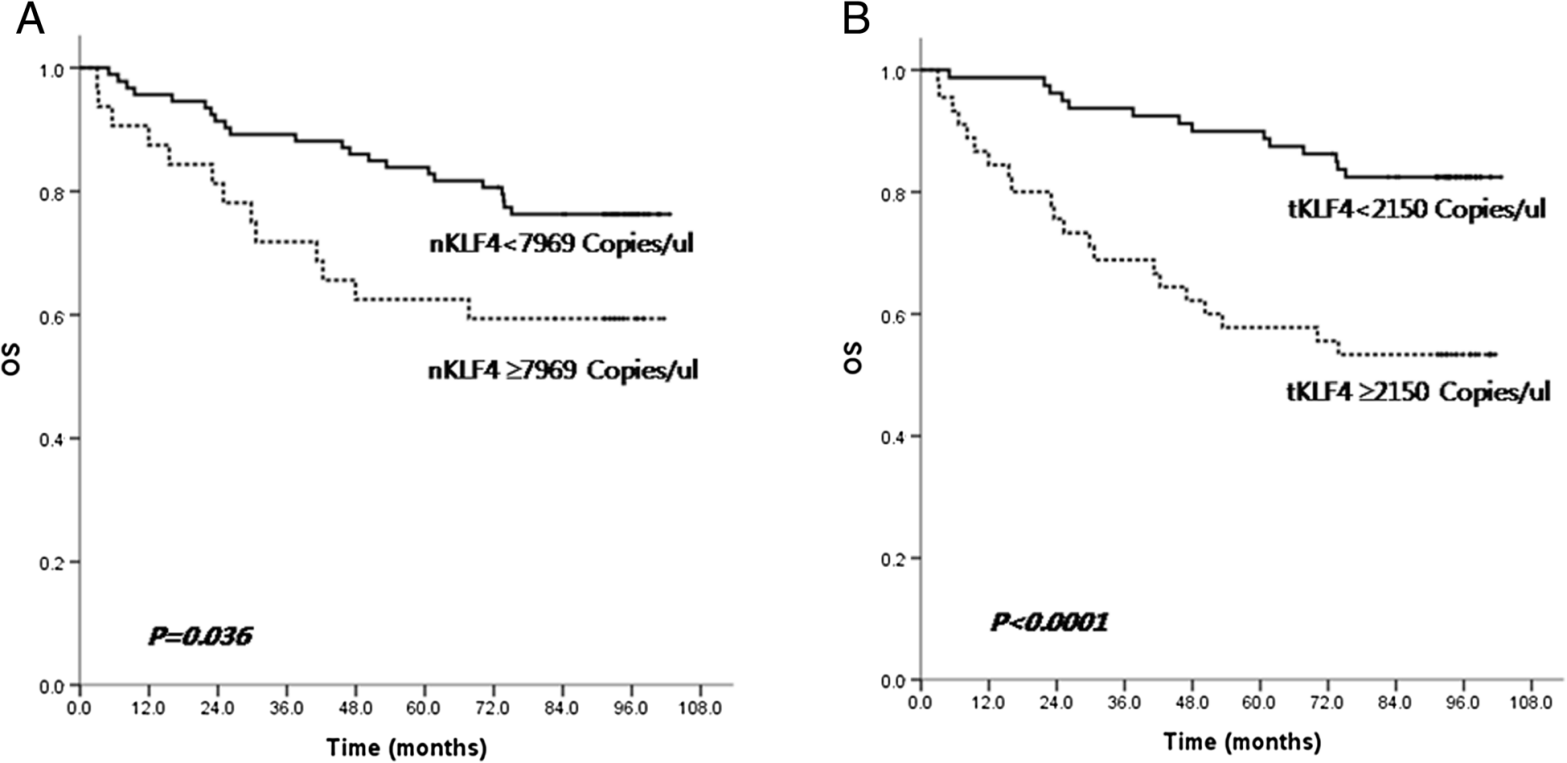
**Kaplan-Meier overall survival (OS) curve of *nKLF4 *and *tKLF4 *expression in all patients. (A)** OS was significantly poorer in patients with high expression level of both *nKLF4* (*P* = 0.036). **(B)** OS was significantly poorer in patients with high expression level of both *tKLF4* (*P* < 0.0001).

Figure 4 shows that OS was significantly poorer in patients with a high expression level of both *nKLF4* and *tKLF4* (*P* = 0.036, *P* <0.0001, respectively). In multivariate analysis, a higher level of *nKLF4* (*nKLF4* ≥ 7696) was an independent risk factor for survival (*P* = 0.005). Other independent risk factors for survival were advanced stage, lymph node metastasis, and poor differentiation.

**Figure 5:**
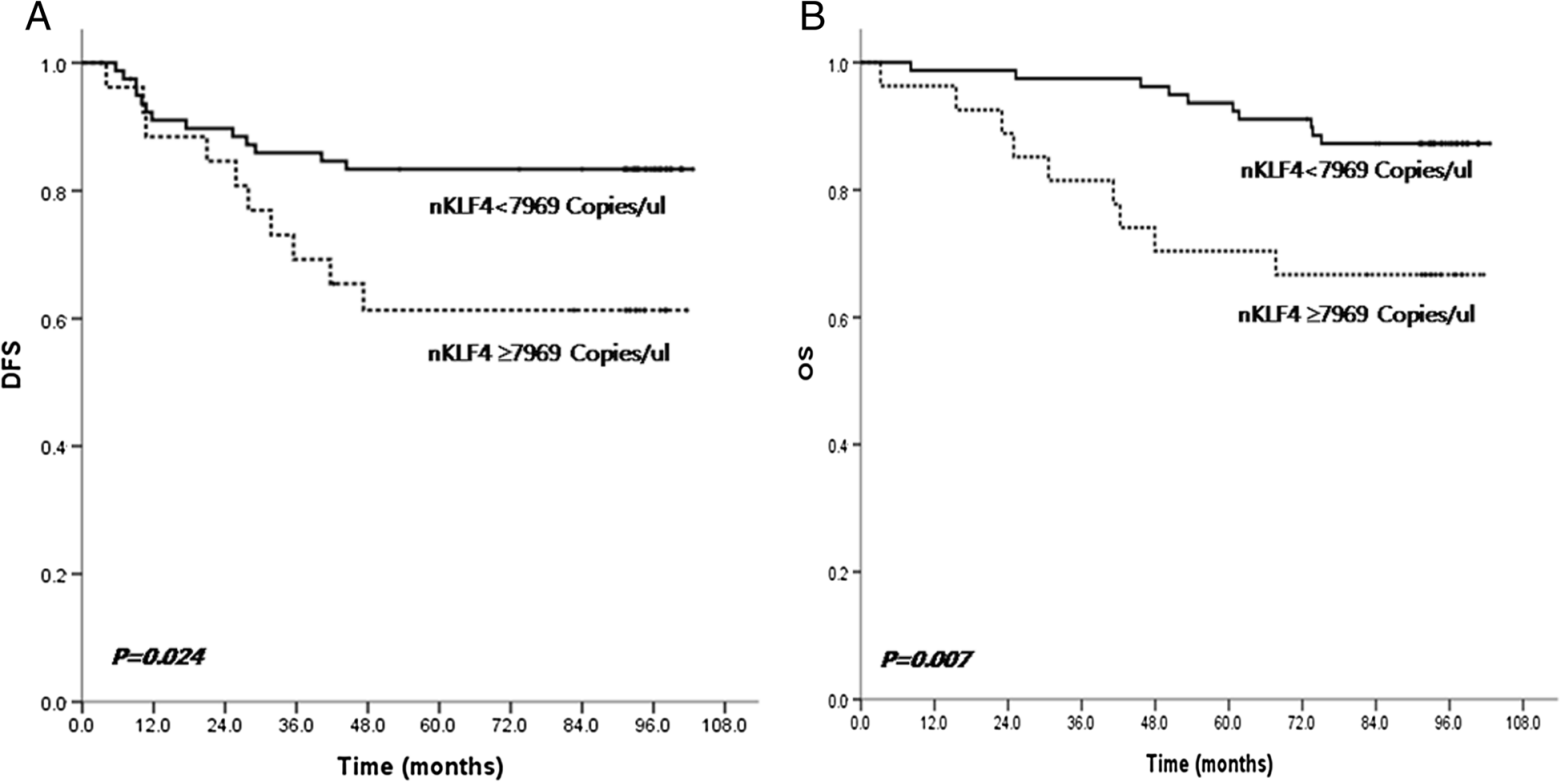
**Kaplan-Meier disease-free survival (DFS) and overall survival (OS) curve of nKLF4 in stage I-III patients. (A)** In patients without distant metastases (stage I-III), those with higher nKLF4 had decreased DFS (*P* = 0.024). **(B)** In patients without distant metastases (stage I-III), those with higher nKLF4 had decreased OS (*P* = 0.007).

In patients without distant metastases (stage I-III), those with higher nKLF4 had decreased DFS and OS (P = 0.024, *P* = 0.007, respectively) (Figure 5). In multivariate models that included age, stage, lymph node status, and nKLF4, only nKLF4 was a significant predictor for DFS (*P* = 0.023) and OS (*P* = 0.011).

## Discussion

Diverse molecular alterations are associated with the initiation and progression of CRC. The zinc-finger transcription factor KLF4 is predominantly expressed in the epithelial cells of the gastrointestinal tract and is an important regulator of intestinal epithelial cell homeostasis and tumorigenesis [18,24,25]. In the few studies that have analyzed the role of KLF4 as a prognostic marker in CRC, a clear relationship has yet to emerge. A recent study showed that loss of KLF4 was an independent predictor of survival and disease recurrence, whereas another study found that loss of KLF4 expression was not associated with survival and disease recurrence [13,22].

In this study, we evaluated the level of KLF4 mRNA expression level using real time RT-PCR in tumors and matched normal tissues. Similar to previous studies, we found a lower expression level of KLF4 in tumors than in normal tissues. Additionally, we observed an elevated KLF4 level in tumors from CRC patients with high KLF4 levels in matched normal tissues. The patients with high KLF4 levels in normal tissues had shorter OS and DFS than those with low KLF4 levels. Therefore, high KLF4 in the normal tissue of individuals appears to be associated with poor prognosis of those individuals with CRC.

A previous study showed that CRC patients who lack KLF4 protein expression in tumors have a poor prognosis [22]. However in our study, patients with high KLF4 mRNA expression in tumors had a worse prognosis than those with low KLF4. A few possibilities exist that may explain this conflicting result. The first is that the different methods used to measure KLF4 expression may produce different results. We measured the level of KLF4 mRNA using real-time RT-PCR, whereas the level of KLF4 protein was detected by immunohistochemistry in the other study [22]. The difference in protein and mRNA expression levels suggests that KLF4 may be regulated at multiple levels. The second possibility is that CRC tissues contained not only tumor cells but also other cells such as surrounding stromal cells. In such a case, elevated KLF4 expression in surrounding stromal cells, despite low KLF4 expression in the tumor cell itself, would skew our analysis, leading us to incorrectly conclude that KLF4 expression in tumors is elevated. We are unaware of any other study that has analyzed the association between KLF4 expression in normal tissues and prognosis. Our study is the first to identify high KLF4 expression in normal tissues as an independent prognostic marker for poor survival and disease recurrence.

Recent studies have revealed that hypermethylation of the KLF4 5′-untranslated region and microRNA-146a (miR-146a), miR-145, or miR-143 expression are associated with altered KLF4 expression in human CRC tissue specimens [18,26,27]. Further studies investigating hypermethylation of the KLF4 5′-untranslated region and microRNA expression will be important to determine how KLF4 expression is regulated in normal and tumor tissues. The most striking aspect of our study is that KLF4 mRNA expression level in normal tissues is an independent predictor of OS and DFS. We identified a significant association between the KLF4 level in both tumor and normal tissue and survival.

## Conclusions

Our data suggests that KLF4 mRNA expression level in both normal and tumor tissue is a potential prognostic marker in patients with CRC. Investigating how the microenvironment influences KLF4 expression and thus tumorigenesis, tumor progression, and prognosis, will be important in future studies.

## Abbreviations

AUC: Area under curve; CRC: colorectal cancer; DFS: Disease-free survival; KLFs: Krüppel-like factors; nKLF4: KLF4 level in normal tissues; OS: Overall survival; ROC curve: Receiver operation characteristic curve; RT-PCR: Real-time reverse transcription-polymerase chain reaction; tKLF4: KLF4 level in tumors; TNM: tumor-node-metastasis.

## Competing interests

The authors declare that they have no competing interests.

## Authors’ contributions

HYL wrote the manuscript. NKK collected and provided the tissues. JBA, SYR, HCC and SJS have contributed part of the experiment, research design, and the data collection. KHP and TSK performed the experiments. SJS oversaw the design of the study, was involved in the critically revised the manuscript. All authors approved the final manuscript.
